# Supplementary material for: Phyto-Computational Intervention of Diabetes Mellitus at Multiple Stages Using Isoeugenol from Ocimum tenuiflorum: A Combination of Pharmacokinetics and Molecular Modelling Approaches
Source: Molecules. 2022 Sep 22;27(19):6222. doi: 10.3390/molecules27196222 (PMC9573403; doi:10.3390/molecules27196222)
Supplement: Supplementary file 1 [file molecules-27-06222-s001.zip › molecules-1886231-supplementary.pdf]

Supplementary Material

Supplementary Table S1: ADMET screening of *O. tenuiflorum* compounds obtained from IMPPAT database

Sl.No	Compound Names	Oral bioavailability (OB≥30%)	Blood-brain barrier (BBB)	Drug half-life (HL<3h)	Lipinski's rule (LR) of five	Intestinal epithelial permeability (Caco-2 cells)	Drug-induced liver injury (DILI)	Clearness (CL>15 ml/min/kg)	Molecular Weight (MW 100~600)	Hydrogen bond acceptor (0~12)	Hydrogen bond donar (0~7)	TPSA (0~140)	PAINS
1	Cyclo(L-Val-L-Leu)	PASS	PASS	0.671	Accepted	-4.711	Negative	5.356	212.150	4	2	58.200	0
2	α-Cubebene	Fail	PASS	0.052	Accepted	-4.408	Negative	18.693	204.190	0	0	0.000	0
3	β-Caryophyllone	Moderate	PASS	0.227	Accepted	-4.750	Negative	14.774	220.180	1	0	17.070	0
4	Phytosterols	PASS	PASS	0.013	Accepted	-4.756	Negative	16.686	414.390	1	1	20.230	0
5	UNII-0V56HXQ8N5	Moderate	Moderate	0.059	Accepted	-4.357	Negative	19.832	204.190	0	0	0.000	0
6	(-)-Alloaromadendrene	PASS	PASS	0.040	Accepted	-4.577	Negative	13.563	204.190	0	0	0.000	0
7	(-)-Camphene	PASS	PASS	0.077	Accepted	-4.463	Negative	9.346	136.130	0	0	0.000	0
8	(-)-*Cis*-Carveol	Fail	PASS	0.378	Accepted	-4.328	Negative	12.624	152.120	1	1	20.230	0
9	(-)-Linalool	PASS	PASS	0.493	Accepted	-4.375	Negative	9.738	154.140	1	1	20.230	0
10	(+)-α-Phellandrene	PASS	PASS	0.617	Accepted	-4.383	Negative	12.660	136.130	0	0	0.000	0
11	(+)-ẟ-Cadinene	Moderate	Moderate	0.051	Accepted	-4.469	Positive	7.421	204.190	0	0	0.000	0
12	(+)-Endo-β-bergamotene	PASS	PASS	0.063	Accepted	-4.466	Negative	16.946	204.190	0	0	0.000	0
13	(1S,2R,4S)-(-)-Bornyl acetate	PASS	PASS	0.243	Accepted	-4.552	Moderate	6.063	196.150	2	0	26.300	0
14	(1S)-1,7,7-Trimethylbicyclo[2.2.1]heptan-2-one	PASS	PASS	0.701	Accepted	-4.582	Negative	13.808	152.120	1	0	17.070	0
15	(E)-β-Farnesene	Fail	Fail	0.156	Accepted	-4.537	Moderate	13.186	204.190	0	0	0.000	0
16	(E)-α-Bisabolene	Fail	Fail	0.092	Accepted	-4.502	Negative	17.581	204.190	0	0	0.000	0
17	(E)-β-ocimene	PASS	PASS	0.678	Accepted	-4.434	Negative	14.171	136.130	0	0	0.000	0
18	1-Octen-3-ol	Fail	Fail	0.672	Accepted	-4.256	Negative	7.650	128.120	1	1	20.230	0
19	1S-α-Pinene	PASS	PASS	0.114	Accepted	-4.303	Negative	15.022	136.130	0	0	0.000	0
20	2,3-Dimethylaniline	PASS	PASS	0.583	Accepted	-4.255	Negative	10.496	121.090	1	2	26.020	0
21	2,5-Dimethoxybenzoic acid	PASS	Moderate	0.885	Accepted	-4.853	Positive	7.488	182.060	4	1	55.760	0
22	3-Carene	PASS	PASS	0.132	Accepted	-4.307	Negative	16.061	136.130	0	0	0.000	0
23	4-Terpineol	PASS	PASS	0.447	Accepted	-4.217	Negative	14.345	154.140	1	1	20.230	0
24	Acetic acid	PASS	PASS	0.791	Accepted	-5.218	Negative	1.609	60.020	2	1	37.300	0
25	AcetyleugeNol	PASS	PASS	0.843	Accepted	-4.453	Moderate	8.457	206.090	3	0	35.530	0
26	α-Fenchene	PASS	PASS	0.099	Accepted	-4.460	Negative	10.559	136.130	0	0	0.000	0
27	α-Humulene	Fail	No	0.095	Accepted	-4.425	Negative	8.432	204.190	0	0	0.000	0
28	α-Terpeneol	PASS	PASS	0.527	Accepted	-4.193	Negative	8.942	154.140	1	1	20.230	0
29	Apigenin	Fail	No	0.856	Accepted	-4.847	Positive	7.022	270.050	5	3	90.900	0
30	Apigenin-7-glucuronide	Fail	No	0.715	Rejected	-6.376	Positive	1.194	446.080	11	6	187.120	0
31	β-Cadinene	Fail	Moderate	0.060	Accepted	-4.392	Negative	17.975	204.190	0	0	0.000	0
32	β-Carotene	Moderate	No	0.076	Rejected	-6.003	Negative	0.229	536.440	0	0	0.000	0
33	β-Caryophyllene	PASS	PASS	0.048	Accepted	-4.517	Negative	9.943	204.190	0	0	0.000	0
34	β -Pinene	PASS	PASS	0.107	Accepted	-4.460	Negative	10.097	136.130	0	0	0.000	0
35	*Bis*-(Acetic acid)	Fail	No	0.998	Rejected	-7.722	Positive	12.869	1700.170	46	25	777.980	1 alert
36	Carotene	PASS	No	0.036	Rejected	-5.634	Negative	0.671	536.440	0	0	0.000	0
37	Carvacrol	Fail	PASS	0.671	Accepted	-4.436	Negative	11.335	150.100	1	1	20.230	1 alert
38	*Cis*-Anethole	PASS	Moderate	0.638	Accepted	-4.440	Negative	11.146	148.090	1	0	9.230	0
39	Decanal	Fail	PASS	0.456	Accepted	-4.551	Negative	5.049	156.150	1	0	17.070	0
40	Dehydro-p-cymene	PASS	PASS	0.568	Accepted	-4.344	Moderate	10.755	132.090	0	0	0.000	0

41	Dipentene	Fail	PASS	0.233	Accepted	-4.320	Negative	11.517	136.130	0	0	0.000	0
42	Estragole	Moderate	Moderate	0.577	Accepted	-4.308	Negative	12.054	148.090	1	0	9.230	0
43	Eucalyptol	PASS	PASS	0.352	Accepted	-4.414	Negative	8.066	154.140	1	0	9.230	0
44	EugeNol	Fail	PASS	0.887	Accepted	-4.373	Negative	14.042	164.080	2	1	29.460	0
45	γ-Selinene	PASS	PASS	0.088	Accepted	-4.577	Negative	13.350	204.190	0	0	0.000	0
46	Geranyl acetate	PASS	PASS	0.506	Accepted	-4.420	Moderate	9.707	196.150	2	0	26.300	0
47	IsoeugeNol	PASS	Moderate	0.880	Accepted	-4.579	Negative	13.435	164.080	2	1	29.460	0
48	L-ascorbic acid	Fail	Fail	0.928	Accepted	-5.917	Positive	9.964	176.030	6	5	114.290	0
49	LiNolenic acid	Fail	Fail	0.710	Accepted	-4.631	Negative	4.877	278.220	2	1	37.300	0
50	Luteolin 7-O-glucuronide	Fail	Fail	0.855	Rejected	-6.471	Positive	1.614	462.080	12	7	207.350	1 alert
51	MethyleugeNol	Moderate	PASS	0.848	Accepted	-4.338	Negative	11.466	178.100	2	0	18.460	0
52	Molludistin	Fail	Fail	0.290	Accepted	-5.776	Positive	3.398	416.110	9	5	149.820	0
53	Myrcene	PASS	PASS	0.453	Accepted	-4.402	Moderate	13.108	136.130	0	0	0.000	0
54	Nerol	Fail	PASS	0.737	Accepted	-4.299	Positive	12.604	154.140	1	1	20.230	0
55	Octadeca-9,12-dieNoic acid	Moderate	Fail	0.628	Accepted	-4.733	Negative	3.327	280.240	2	1	37.300	0
56	OctadecaNoate	Fail	Fail	0.476	Accepted	-5.068	Negative	2.425	284.270	2	1	37.300	0
57	Oleic acid	Moderate	Fail	0.546	Accepted	-4.922	Negative	2.573	282.260	2	1	37.300	0
58	Orientin	Fail	Fail	0.724	Rejected	-6.208	Positive	5.042	448.100	11	8	201.280	1 alert
59	Palmitic acid	Fail	Fail	0.610	Accepted	-5.027	Negative	2.377	256.240	2	1	37.300	0
60	Thymol	Fail	PASS	0.682	Accepted	-4.387	Negative	9.444	150.100	1	1	20.230	0
61	Ursolic acid	Moderate	PASS	0.017	Accepted	-5.221	Negative	3.671	456.360	3	2	57.530	0
